# To Give or Not to Give Inhaled Antibiotics: Heterogeneous Effects of Inhaled Antibiotics in Ventilated Patients in the Intensive Care Unit

**DOI:** 10.7759/cureus.108645

**Published:** 2026-05-11

**Authors:** Tina H Dao, Jason Harhay, Rida N Khan, Jonathan J James, Nathan A Summers

**Affiliations:** 1 Department of Medicine, University of Tennessee Health Science Center, Memphis, USA; 2 Department of Medicine, Division of Infectious Diseases, University of Tennessee Health Science Center, Memphis, USA; 3 Department of Medicine, Division of Critical Care, Kansas University Medical Center, Kansas City, USA

**Keywords:** antibacterial synergy, antibiotic usage, inhaled drugs, intensive care unit (icu), ventilator-associated pneumonia

## Abstract

Background

Ventilator-associated pneumonia (VAP) is one of the most common causes of morbidity and mortality in the intensive care unit (ICU). Inhaled antibiotics, which can directly target lung pathogens, have been used to treat VAP in the ICU for the past few decades. However, data on their efficacy and safety profiles remain limited. The primary objective of this study was to assess the effects of inhaled antibiotics on clinical outcomes for patients with VAP.

Methods

In this study, we conducted a retrospective cohort study including all patients at our Regional One Health in Memphis, TN, who were diagnosed and treated for VAP. Our overall objective was to assess the treatment efficacy of inhaled antibiotics as adjunct therapy for VAP on overall hospital length of stay (LOS), days on the ventilator, days in the intensive care unit, and respiratory culture clearance from antimicrobial agents.

Results

There were 383 ventilated patients diagnosed with VAP included for analysis, and 52 of those received inhaled antibiotics. The use of inhaled antibiotics for ventilated patients in the ICU was associated with shorter hospital LOS (incidence rate ratio (IRR)=0.89, 95% confidence interval (CI) 0.84-0.94, p<0.001) but no change in ICU LOS (IRR=1.05, 95% CI 0.99-1.11, p=0.11). There was no statistically significant association between the use of inhaled antibiotics and ventilator duration or respiratory culture clearance.

Conclusions

These findings suggest that inhaled antibiotics are likely not required for every ventilated patient in the ICU; however, more studies are needed to clarify their role in the treatment of VAP in similar patient populations.

## Introduction

Multidrug-resistant (MDR) Gram-negative organisms are becoming an increasingly difficult barrier to the treatment of patients, particularly when treating patients who are critically ill. These resistant strains include “ESKAPE” pathogens (*Enterococcus faecium*, *Staphylococcus aureus*, *Klebsiella pneumoniae*, *Acinetobacter baumannii*, *Pseudomonas aeruginosa*, and *Enterobacter* species). Currently, the development of new antibiotics cannot compete with the rate of emerging resistance [1]. As such, alternative methods to combat multidrug-resistant species have been employed. While inhaled antibiotics have been available for use since the 1940s for both active and chronic lung infections, the usage of inhaled antibiotics as an adjunctive therapy in the treatment of ventilator-associated pneumonia (VAP) has recently increased [2,3]. There are currently three FDA-approved inhaled antibiotics-aztreonam, colistin, and tobramycin. Historically, these antibiotics have been used to treat chronic infections related to patients with cystic fibrosis (CF) and chronic *P. aeruginosa* infections. Moreover, injectable versions of gentamicin, tobramycin, amikacin, ceftazidime, and amphotericin have been nebulized for off-label use for the management of non-CF bronchiectasis, drug-resistant nontuberculous mycobacterial infections, ventilatory-associated pneumonia, and post-transplant airway infections [4-7]. The inhaled antibiotics can provide high concentrations of the drug into the lung that could not be achieved by systemic antimicrobials without systemic side effects [2]. While the theory behind inhaled antibiotics seems encouraging, there is limited data to support their use as a routine modality since very few randomized controlled trials that have examined their efficacy exist.

In this study, we performed a retrospective cohort study to investigate the effects of inhaled antibiotics within the intensive care units (ICUs) at a local hospital as it pertains to non-CF infections, particularly those with VAP. Our overall objective was to assess the treatment efficacy of inhaled antibiotics as adjunct therapy for VAP on overall hospital length of stay (LOS), days on the ventilator, days in the intensive care unit, and respiratory culture clearance from antimicrobial agents. These results would provide insights into the efficacy of inhaled antibiotic agents in the treatment of VAP in the ICU.

This article was previously presented as a poster at the 2025 American Thoracic Society International Conference.

## Materials and methods

Study design and inclusion and exclusion criteria

In this respective analysis, all ventilated patients with respiratory infections at the Regional One Health (ROH) Medical Center in Memphis, TN, USA, from July 1, 2021, through July 1, 2023, were included for chart review. Regional One Health serves as the region’s safety net hospital and is the region’s only level one trauma center and burn center. It has four ICUs: a burn ICU, a trauma ICU, a medical ICU, and a long-term acute care hospital (LTACH) ICU, all of which were included in the analysis. After obtaining approval from the University of Tennessee Health Science Center (UTHSC) institutional review board and ROH research committee, the data team at ROH identified all cases of ventilator-associated pneumonia (VAP) by International Classification of Diseases (ICD)-9 or ICD-10 codes. Patients were excluded if, after chart review, they were found not to be diagnosed or treated for VAP.

Data collection

A list of all potential patients with VAP was created by the data team at ROH using ICD-9 or ICD-10 codes. Each patient was given a participant ID, which was then linked to a standardized case report form (CRF) that then only contained de-identified information. The CRF was used to manually abstract chart information, including patient demographics, treatment interventions, and health outcomes. Only patients who were documented to have been diagnosed and treated for VAP were included in the main analysis. Patients were classified as either receiving inhaled antibiotics or not based on documented administration of at least one dose of an inhaled antimicrobial. Selection, dosing, and frequency of administration of antimicrobials were left to the prescribing physicians and clinical pharmacists. Clearance of respiratory cultures was determined to be achieved if subsequent respiratory cultures were obtained after initiation of treatment and the new respiratory cultures did not grow the same pathogen originally being treated.

Statistical analysis

Our primary outcomes included ICU LOS (days), hospital LOS (days), ventilator duration (days), and respiratory culture clearance. All data analyses were performed on the de-identified CRF. Univariate analyses were conducted using Fisher's exact, Pearson's Chi-squared, or Wilcoxon rank-sum test. Multivariable analysis was conducted using a Poisson regression for hospital LOS, ICU LOS, and ventilator duration, and logistic regression for respiratory culture clearance. All models were developed using stepwise selection with the Akaike Information Criterion, balancing goodness-of-fit with model simplicity. All analyses were conducted using R version 4.4.0 (R Foundation for Statistical Computing, Vienna, Austria).

## Results

A total of 383 ventilated patients were included in this retrospective study, and 52 of them received inhaled antibiotics. The inhaled antibiotics that the patients received were amikacin, tobramycin, and colistin. The median age was 53.6 years; 134 (35%) identified as Caucasian, 225 (59%) identified as African American, and the remaining identified as other; 275 (72%) identified as male; 252 (66%) met sepsis criteria. 253 (66%) were diagnosed with VAP, and 41 of those received inhaled antibiotics (Table 1).

**Table 1:** Patient characteristics.

Characteristics	All ventilated patients (N=383)	Inhaled antibiotics (N=52)	No inhaled antibiotics (N=331)
Age, years, median (IQR)	54 (34-67)	58 (36-68)	52 (34-66)
Race			
Caucasian, n (%)	134	18 (13%)	116 (87%)
African American, n (%)	225	34 (15%)	191 (85%)
Other, n (%)	21	0 (0%)	21 (100%)
Unknown, n (%)	3	0	3
Ethnicity			
Non-Hispanic, n (%)	369	52 (14%)	317 (86%)
Hispanic, n (%)	13	0 (0%)	13 (100%)
Unknown, n (%)	1	0	1
Gender			
Male, n (%)	275	39 (14%)	236 (86%)
Female, n (%)	108	13 (12%)	95 (88%)
Sepsis	252	41 (16%)	211 (84%)
Unknown	1	0	1
Charlson Comorbidity Index, median (IQR)	2.00 (0-3)	2.00 (0-3.5)	2.00 (0-3)
Pneumonia type			
Community-acquired, n (%)	60	3 (5%)	57 (95%)
Hospital-acquired, n (%)	63	8 (13%)	55 (87%)
Ventilator-associated, n (%)	253	41 (16%)	212 (84%)
Unknown, n (%)	7	0	7

We compared hospital LOS, ICU LOS, ventilator duration, and respiratory culture clearance between ventilated patients who received at least one dose of inhaled antibiotics and those who did not. The use of inhaled antibiotics for ventilated patients in the ICU was associated with a trend toward longer ICU LOS (IRR=1.05, 95% confidence interval (CI) 0.99-1.11, p=0.11) but shorter hospital LOS (incidence rate ratio IRR=0.89, 95% CI 0.84-0.95, p<0.001) (Table 2). Respiratory clearance was achieved in 92 (24%) ventilated patients, of which 11 received inhaled antibiotics. Overall, there was no statistically significant association between the use of inhaled antibiotics and ventilator duration or respiratory culture clearance.

**Table 2:** Primary outcome. IRR: incidence rate ratio; CI: confidence interval; LOS: length of stay *Calculated by multivariable analysis using a Poisson regression.

Outcome	Inhaled antibiotics vs. no inhaled antibiotics		
	IRR	95% CI	p-value*
Ventilator duration	1.03	0.96-1.1	0.4
ICU LOS	1.05	0.99-1.11	0.11
Hospital LOS	0.89	0.85-0.95	<0.001
Respiratory culture clearance	0.81	0.35-1.80	0.6

## Discussion

In this retrospective study, we aimed to determine whether the use of inhaled antibiotics was effective at treating VAP in the ICU and eradicating the causative pathogens of VAP. While there was a slight improvement in overall hospital LOS with the use of inhaled antibiotics, there were no significant changes in ICU LOS, ventilator duration, or clearance of respiratory cultures. These results suggest minimal clinical benefit of inhaled antibiotics when treating VAP in the general patient population.

Inhaled antibiotics facilitate the delivery of high concentrations of antibiotics directly to the lungs without inducing systemic adverse effects. Several studies have shown potential benefits of inhaled antibiotics in critically ill patients [8-10]. Lu and his colleagues compared the efficacy of inhaled versus intravenous amikacin and ceftazidime in patients with *P. aeruginosa* VAP. Seventy percent of patients who received inhaled amikacin and ceftazidime were successfully treated; however, the result was not statistically significant when compared to the intravenous group [11]. Later, Tulli et al. conducted a meta-analysis showing that inhaled colistin was found to be non-inferior to intravenous standard of care treatment for VAP [12]. Another meta-analysis and systematic review conducted by Valachis et al. also observed improved clinical response and bacterial clearance with adjunctive inhaled colistin compared to intravenous; however, inhaled colistin did not reduce mortality [13].

Similar to previous studies, our study showed that inhaled antibiotics exerted heterogeneous effects on clinical outcomes [8-13]. The ventilated patients in our study who received inhaled antibiotics had a shorter hospital LOS but a trend toward a longer ICU LOS. Furthermore, the addition of inhaled antibiotics did not significantly affect ventilator duration or clearance of respiratory pathogens. One possible explanation is that although inhaled antibiotics facilitate delivery of high concentrations of the antibiotics directly to the lungs and potential, they may not be able to reach poorly aerated, consolidated lung parenchyma during a pneumonia to exert their antimicrobial effects. It is also not clear if the heterogeneous effects in this work suggest an overall lack of efficacy of inhaled antibiotics or whether there may be target populations who benefit from this intervention.

There were several limitations to our work. As a retrospective cohort study, it is possible there were unknown, unmeasured factors confounding the relationship between inhaled antibiotics and clinical outcomes, or even affecting whether or not inhaled antibiotics were started at all. Although our medical center has several different types of ICUs (burn, trauma, medical, and LTACH) that were included in the analysis, being a single-center study somewhat limits generalizability. Finally, while we observed heterogeneous clinical outcomes related to inhaled antibiotics, the overall effect sizes were very small with a questionable clinical impact. This may suggest that there may be specific patient populations that may benefit most from inhaled antibiotics, but we were limited in identifying these due to our smaller sample size. While we attempted to control for many of these factors in our multivariable analysis, larger, multicenter randomized controlled trials are needed to better address the utility of inhaled antibiotics.

## Conclusions

In conclusion, we observed heterogeneous effects of inhaled antibiotics on hospital LOS, ICU LOS, ventilator duration, and respiratory culture clearance in ventilated patients in the ICU, with relatively small effect sizes for each outcome. Our data suggest that inhaled antibiotics are unlikely to provide benefit for most ventilated patients in the ICU who are being treated for VAP. Further study is needed to identify if specific patient populations may benefit from inhaled antibiotics.
